# Hemodynamic and morphological changes of the central retinal artery in myopic eyes

**DOI:** 10.1038/s41598-022-11087-x

**Published:** 2022-05-02

**Authors:** Mei Zhao, Andrew Kwok-Cheung Lam, Michael Tin-Cheung Ying, Allen Ming-Yan Cheong

**Affiliations:** 1 School of Optometry, Faculty of Health and Social Science, The Hong Kong Polytechnic University, Hung Hom, Kowloon, Hong Kong China; 2 Research Centre for SHARP Vision, The Hong Kong Polytechnic University, Hung Hom, Kowloon, Hong Kong China; 3 Centre for Eye and Vision Research, Kowloon, Hong Kong China; 4 Department of Health Technology and Informatics, The Hong Kong Polytechnic University, Hung Hom, Kowloon, Hong Kong China

**Keywords:** Biomarkers, Diseases, Health care, Medical research, Risk factors

## Abstract

Due to excessive elongation of the eyeball, myopia-related vascular abnormalities are frequently observed in the central retinal artery (CRA) and its intraretinal branches. In addition to inconsistency in previously reported findings, hemodynamic (reduced flow velocity, increased vascular resistance) and morphological changes (narrower vessel diameter) were usually studied separately. This cross-sectional study evaluated the hemodynamic and morphological characteristics concurrently in a large sample of healthy myopes, by using the color Doppler ultrasound and adaptive optics retinal camera. Results showed that the retrobulbar segment of CRA had a tendency of slightly reduced flow velocity in eyeballs with longer axial length, but the correlation was not significant after adjusting for the multiple correlations. Vascular resistance was not affected by the axial elongation. With respect to the intraretinal branches, no significant changes in longer eyes of total diameter or lumen diameter were observed, while both the wall thickness and the wall cross-sectional area were significantly increased, but only a marginally increase in the wall to lumen ratio was found with increasing axial length. This implies some potential small artery remodeling in the intraretinal CRA branches. Overall, blood supply of the inner retina in healthy young myopes is likely to be maintained. Additionally, morphological parameters of vascular microstructure could be potential biomarkers to monitor myopia progression and understand myopia-related vascular abnormalities in future studies.

## Introduction

The soaring prevalence of myopia and high myopia undoubtedly imposes huge concern in our society. As a “twenty-first century public health issue”^1^, what myopia and high myopia bring to humans is far beyond a pair of correcting spectacles, but an increased risk of developing sight-threatening pathologies, including glaucoma, retinal detachment, and myopic macular degeneration. As suggested by the International Myopia Institution^2^, refractive error with spherical equivalent of ≤ −6.00 D with relaxed ocular accommodation is defined as high myopia, which is characterized as excessive eyeball elongation in most circumstances.

Extensive studies have revealed associations between excessive eyeball elongation and various vascular abnormalities in myopic eyes (refer to Zhao et al. for a recent review^3^). At the retinal level, myopic eyes showed reduced capillary perfusion at foveal^4,5^ and peripapillary regions^6,7^, enlarged foveal avascular zone^8,9^, narrower retinal vessels^10^, and higher percentage of peripheral avascularity^11^.

The retina is particularly vulnerable to ischemia because of the high-oxygen demands and relatively sparse nature of the retinal vasculature^12^, especially for the inner layers (up to the inner part of the inner nuclear layer), which are mainly supplied by the central retinal artery (CRA). The CRA branches from the ophthalmic artery and pierces into the optic nerve sheath around 10 mm posterior to the globe^13^. After entering the eyeball, it first divides into the superior and inferior branches, and each of these further branches temporally and nasally to perfuse different regions of the inner retina^13^.

Myopia-related vascular changes occur to the retrobulbar CRA and its intraretinal branches. In the retrobulbar segment, color Doppler ultrasound, a commonly used technique for examining the retrobulbar circulation, disclosed impaired CRA hemodynamics in highly myopic eyes, as reflected by reduced blood flow velocities^14–17^ and increased vascular resistance^15,18^. The compromised CRA hemodynamics have been associated with increased severity of myopic degenerative changes^19^. However, inconsistent findings have been reported by other studies using similar techniques. Galassi et al.^20^ reported no difference between highly myopic eyes, with axial length (AL) > 27.5 mm, and emmetropic controls, with AL ≤ 25.5 mm, in either velocities or resistive index. Dimitrova et al.^17^ observed a negative correlation between axial length and resistive index. Despite numerous studies investigating the effect of axial elongation on CRA hemodynamics, the findings remain inconclusive. One possible reason is the high individual variabilities of hemodynamic measures and relatively low repeatability of these measures^21^.

Shimada et al.^10^ measured the flow velocity and vessel diameter of the major retinal artery (the superotemporal or inferotemporal CRA branch) at the point after piercing into the eyeball but before its first branching. They reported narrower branch diameter and unchanged flow velocity, which together indicated a reduced blood flow in high myopes (refraction < − 8.0 D) compared with mild myopes (refraction between − 8.0 to − 3.0 D) and emmetropes (refraction within ± 3.0 D). Comparing with highly myopic eyes with normal or tessellated fundus, the retinal arteries were further narrowed in the myopic eyes with advanced myopic maculopathies, including posterior staphyloma, lacquer cracks, and choroidal atrophy^22,23^.

As a result of an impaired vascular system, the oxygen supply of the retina may be altered. In myopic eyes, the arterial and venous oxygen saturation were analyzed using a spectrophotometric retinal oximeter. Briefly, the principle is that the light absorbance of vessels changes with oxygen saturation (i.e. concentration of the deoxyhemoglobin and oxyhemoglobin). The device can generate a color-coded map based on the light absorbance of each vessel when viewed at specific light wavelengths, separating the artery from the vein. In healthy populations, the oxygen saturations of the retinal artery and vein were about 92% and 56%, respectively^24^. Highly myopic eyes had decreased artery oxygen saturation but venous saturation was unchanged^22,25,26^, suggesting that relative oxygen consumption, the difference between arterial and venous oxygen saturation, might be decreased in highly myopic eyes^22^.

Altered oxygen consumption could lead to functional abnormality of the inner retina. In fact, myopia-related functional loss of the inner retina has been revealed by several electrophysiology studies^27–30^. Man, et al.^31^ investigated the relationships between axial length, retinal function (by multifocal electroretinography (mfERG)), and relative oxygen consumption in healthy individuals. Significant associations among increasing AL, impaired central and peripheral retinal function (i.e. decreased mfERG amplitude), and reduced oxygen consumption were identified. Path analysis further revealed that reduced oxygen consumption in longer eyes was indirectly mediated by the decrease in retinal function, indicating that retinal metabolic demands were decreased in longer eyes due to a loss of functional neurons.

Although myopia-related morphological and hemodynamic changes of the CRA have been studied extensively, usually these changes were reported separately. It remains uncertain whether the eyeball elongation affects the retrobulbar or the intraretinal vessels asynchronously or synchronously. Understanding this relationship helps to reveal the distribution and effect of mechanical stretching force at different segments of myopic eyeballs, and further contributes to the knowledge of myopic traction maculopathies. From the anatomy perspective, the retrobulbar segment of the CRA is surrounded and protected by the optic nerve sheath, so it is less likely to be altered due to eyeball elongation. Although narrower intraretinal arteries in myopic eyes were reported by previous studies^10,22,23^, insufficient information regarding the morphological changes of retinal vessels were provided due to the limited resolution of conventional imaging techniques. The narrower artery could be a result of a thinner vessel wall or reduced lumen or both. Thus, the overall vessel diameter might be inadequate to monitor the subtle changes in the vascular microstructures.

This study aimed to investigate the hemodynamic and morphological changes of the CRA in young healthy myopic adults, using color Doppler ultrasonography (CDU) and an adaptive optics (AO) fundus camera. CDU has long been used to study the retrobulbar circulation in various ocular conditions. AO retinal imaging offers a more precise way to measure vessel caliber by detecting and correcting the wavefront aberrations of human eyes. Its high resolution for distinguishing lumen from vessel wall makes it a better tool to detect potential vascular changes in myopic eyes.

## Methods

This observational, cross-sectional study was conducted in the Optometric Research Clinic, School of Optometry, and the Ultrasound Laboratory, Department of Health Technology and Informatics, The Hong Kong Polytechnic University after prior approval from the University Human Subjects Ethics Sub-committee. All the study procedures adhered to the tenets of the Declaration of Helsinki. A written, informed consent explaining the study procedure and the potential risks was obtained from each participant.

### Clinical examinations

Healthy young adults aged between 18 and 40 years were recruited. A preliminary optometric examination was conducted to confirm subject’s eligibility and collect demographic data. Subjective refraction was performed with the best-corrected distance visual acuity (BCVA) recorded using an electronic LogMAR visual acuity chart (Test Chart Xpert 3Di, Version 12 08 13, Thomson Software Solutions, UK). Five consecutive measures of axial length (AL) were obtained by IOL Master (Carl Zeiss, Germany), and the average was used for data analysis. Three readings of intraocular pressure (IOP) were obtained by non-contact tonometry (CT-80 Computerized Tonometer, Topcon, Japan). External eye health was examined by slit-lamp biomicroscopy. Fundus photography and optical coherence tomography (OCT, Cirrus, Carl Zeiss) were performed to confirm the internal ocular condition of the posterior pole. An Optic Disc Cube 200 × 200 protocol was used to scan the peripapillary region. The OCT device automatically identified and outlined the disc center and contour in the infrared fundus image, which was later used as the reference for image analysis (see more details in the “Adaptive optics (AO) fundus camera” section). Subjects with extremely high myopia (spherical equivalent, SE ≤ − 9.00D) went through a static automated perimeter examination (Humphrey Field Analyzer, Swedish Interactive Testing Algorithm, 24-2 SITA fast) additionally to rule out any visual field defects. Myopia-related retinal abnormalities, such as staphyloma, chorioretinal atrophy, retinal degenerations, were not used as exclusion criteria provided that these abnormalities did not affect the central vision.

Subjects with any of the following conditions were excluded: (1) systematic or ocular diseases, which could affect ocular blood circulation (e.g. diabetes mellitus, hypertension, choroidal neovascularization); (2) glaucomatous visual field defects with associated thinning of the retinal nerve fiber thickness; (3) media opacities which could affect the image quality; (4) history of ocular surgery (e.g. refractive surgery, laser iridotomy) or injury; (5) LogMAR BCVA > 0.00 of both eyes; (6) abnormal intraocular pressure (> 21 mmHg); (7) anisometropia (SE difference of two eyes > 2.50D); and (8) history of smoking. All participants were asked to avoid alcohol and caffeine intake 12 h before the examinations to prevent any potential effects on ocular circulation.

### Adaptive optics (AO) fundus camera

Only one eye of each subject was randomly selected for AO imaging in a dark room with natural pupil. For eyes with a small pupil size, which blocked the imaging area, a drop of Mydrin-P ophthalmic solution (phenylephrine hydrochloride 0.5% and tropicamide 0.5%) was administrated. The temporal branch of the CRA (either the superior or inferior branch, depending on the visibility) was imaged by the AO retinal camera (rtx1; Imagine Eyes, Orsay, France) using the “retinal vessels” mode. A series of 4° × 4° (~ 1.2 mm × 1.2 mm) AO images were captured, under the guidance of the internal fixation target, to cover a vessel length of about 1.5-disc diameter (DD) from the optic disc margin. The area of interest was cross-checked with the fundus photo.

**Figure 1:**
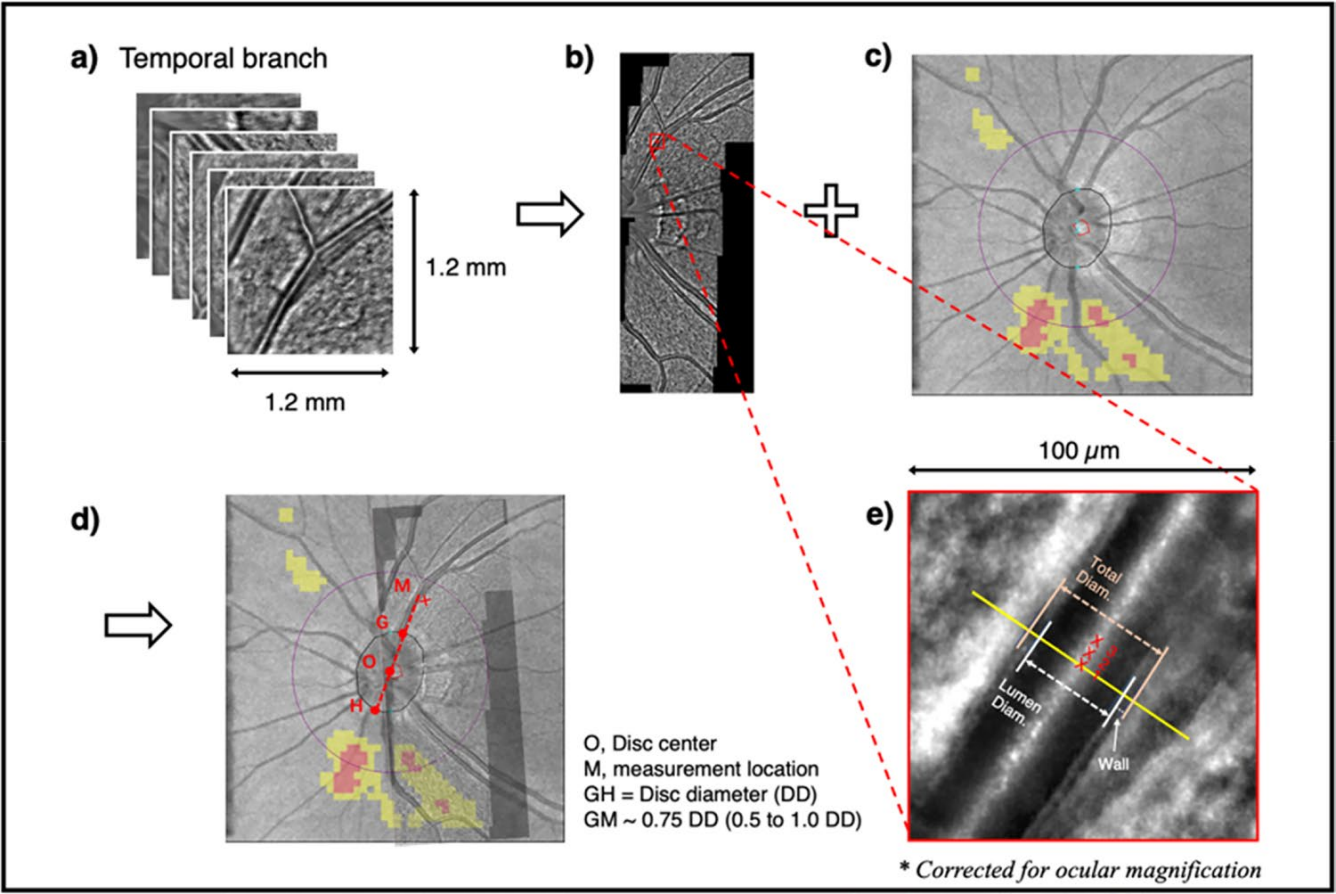
Image processing to obtain the morphological parameters of the temporal branch of the central retinal artery. Raw images (**a**) from the adaptive optics fundus camera were automatically stitched, using i2k Retina Pro software (Version 3.1.0, DualAlign, LLC, Clifton Park, NY), to create the montage (**b**), which was later manually superimposed onto the infrared fundus image (**c**) to mark the measurement location (**d**), using Microsoft PowerPoint for Mac (Version 16.59, Microsoft Corporation, Washington, USA) and Fiji (Version 2.1.0/1.53c, Open source image processing package^32^). The center and contour of the optic disc (point O) was automatically identified by the imaging software. The actual measurement location (point M) was about 0.75 disc diameter from the disc margin (distance between point G and H). Three adjacent measurements (**e**) were taken after correcting ocular magnification by the axial length. Total diameter, lumen diameter and wall thickness of the retinal artery were measured by the AOdetect Artery (version 2.0b17, Imagine Eyes, Orsay, France).

The procedure of image processing is illustrated in Fig. 1. Multiple AO images were stitched by an embedded auto-montage software (i2k Retina Pro, Version 3.1.0, DualAlign, LLC, Clifton Park, NY). The AO montage was manually superimposed onto the infrared fundus images (extracted from the OCT scan) to mark the measurement location, using Microsoft PowerPoint for Mac (Version 16.59, Microsoft Corporation, Washington, USA) and Fiji (Version 2.1.0/1.53c, Open source image processing package^32^). The AO montage was then analyzed by another customized software (AOdetect Artery, Version 2.0b17, Imagine Eyes, Orsay, France) to obtain the morphological parameters, including total diameter, lumen diameter, wall thickness, wall to lumen ratio, and wall cross sectional area of the retinal artery. The axial length was inputted to correct for the ocular magnification. The measurement started from the superior/inferior temporal branch at a location about 0.75 DD oblique from the disc margin (Fig. 1). Measurement cursor could slightly move closer or further away if the target location was not measurable, but it always fell into a range of 0.5 to 1 DD oblique. Three adjacent measurements were obtained (Fig. 1e), and their average was used for data analysis.

### Color Doppler ultrasound (CDU)

Color Doppler ultrasonography was arranged in a separate visit without pupil dilation. The data collection was conducted at an approximately same time point as the AO imaging (two mornings or two afternoons within the same week) to reduce diurnal fluctuation of blood circulation. Subjects were required to lie supine and rest for at least five minutes to stabilize the hemodynamic condition before data collection. Two repeated measures of systolic (SBP), diastolic blood pressure (DBP), and pulse rate were recorded by an electronic sphygmomanometer on the left arm. Mean arterial pressure (MAP) and ocular perfusion pressure (OPP) were determined as follows: MAP = DBP + 1/3 (SBP − DBP); OPP = 2/3 MAP − IOP.

**Figure 2:**
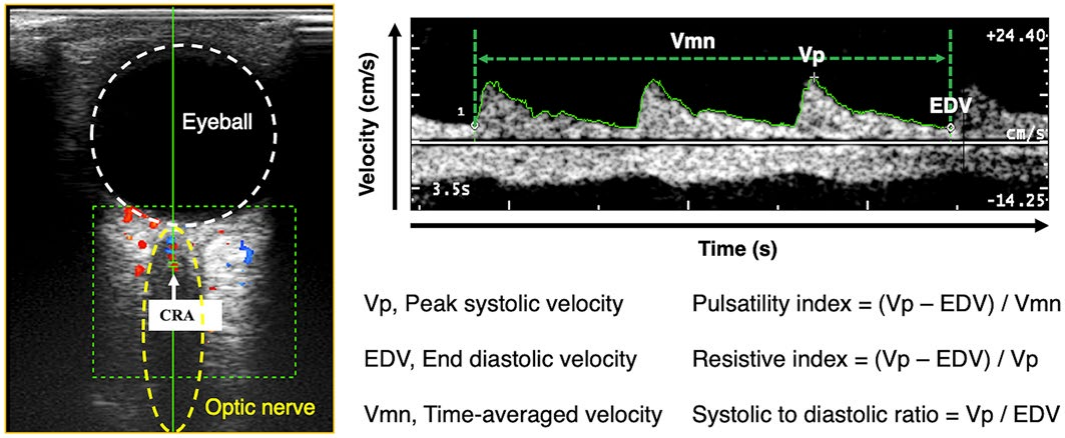
Measurement location and typical waveform of the central retinal artery (CRA) in color Doppler ultrasonography. The locations of the eyeball and optic nerve are outlined by the white circle and yellow ellipse, respectively. Blood flow signals (blue and red areas) appear within the green rectangle. Hemodynamic parameters, including peak systolic velocity (Vp), end diastolic velocity (EDV), time-averaged mean velocity (Vmn), pulsatility index (PI), resistive index (RI) and systolic / diastolic ratio (S/D), were calculated for each triple-waveform.

Retrobulbar hemodynamics of the central retinal artery (CRA) were measured by color Doppler ultrasonography (MyLab™ Twice ultrasound system, Esaote Genoa, Italy). The probe (3–11 MHz linear transducer, LA322E) was covered by a sufficient amount of coupling gel and gently positioned on the closed upper eyelid. The subject was placed in a supine position and instructed to “look straight” while closing the eyes. The optic nerve was identified under the transverse scan of B-mode ultrasound to locate the CRA, which should lie in the middle of the optic nerve (Fig. 2). Once the location was confirmed, color Doppler mode was used to show the color-coded blood flow signals. The sample volume was placed just behind the lamina cribrosa, and spectral Doppler mode was activated to record the blood flow waveforms. Three recordings were obtained, and each recording was completed after three rhythmic waveforms were captured. The built-in program automatically traced the waveforms and calculated six hemodynamic parameters (Fig. 2), including peak systolic velocity (Vp), end diastolic velocity (EDV), time-averaged velocity (Vmn), resistive index (RI), pulsatility index (PI) and systolic to diastolic ratio (S/D). A repeated session of CRA data collection was conducted one hour after the primary session to examine the test–retest repeatability for 30 subjects.

### Statistical analysis

The IBM-SPSS 26 and GraphPad Prism 9.2 were used for data analysis and visualization. Data distribution was examined by the Shapiro–Wilk test, and skewed data were tested by nonparametric tests. Gender differences was tested by independent t-test (or Mann–Whitney test). The test–retest repeatability of hemodynamic parameters was evaluated by the paired t-test (or Wilcoxon signed rank test), the intra-class correlation coefficient and the coefficient of variation. Difference between morphological parameters obtained from the superior and inferior temporal branches were examined by the paired t-test (or Wilcoxon signed rank test). Bivariate Spearman correlation analyses between axial length and other parameters were reported, and univariate linear regression was run for significant correlations. Bonferroni correction was used to adjust p-values in multiple testing. A two-tailed p-value less than 0.05 was considered as statistically significant.

## Results

Demographic information of 118 eyes from 118 subjects (75 females and 43 males) are summarized in Table 1. Age-matched female and male participants were similar in axial length (AL), spherical equivalent (SE), best-corrected visual acuity, intra-ocular pressure, and pulse rate. Blood pressure related parameters, including SBP, DBP, MAP, OPP, were higher in male subjects. This population had a SE ranged from + 1.00 D to − 15.38 D, including 51 eyes with high myopia (SE ≤ -6 D), 30 eyes with moderate myopia (− 6 D < SE ≤ − 3 D) and 37 eyes with low myopia/emmetropia (SE > − 3D). The axial length of 118 eyes extended from 21.99 to 31.28 mm, which was consisted by 50 eyes with an AL ≥ 26 mm, 47 eyes with an AL between 24 and 26 mm, and 21 eyes with AL ≤ 24 mm. Among the 118 eyes, 110 eyes had a normal or tessellated fundus only, 8 eyes with patchy atrophy near the optic disc were categorized as pathological myopia according to the META-PM grading system^33^.

**Table 1:** Descriptive statistics of the demographic information, hemodynamic and morphological characteristics.

Demographic information	Overall (N = 118)	Female (N = 75)	Male (N = 43)	P value
Age (year)	25.91 ± 5.77	25.64 ± 5.77	26.37 ± 5.81	0.463
Axial length (mm)	25.80 ± 1.83	25.67 ± 1.70	26.02 ± 2.02	0.322
Spherical equivalent (Diopter)	− 5.28 ± 3.74	− 5.68 ± 3.57	− 4.58 ± 3.98	0.127
Best-corrected visual acuity (LogMAR)	− 0.06 ± 0.06	− 0.05 ± 0.06	− 0.07 ± 0.06	0.175
Intraocular pressure (mmHg)	15.23 ± 2.61	15.20 ± 2.55	15.26 ± 2.73	0.905
Systolic blood pressure (mmHg)	108.29 ± 11.56	103.68 ± 10.38	116.33 ± 8.85	** < 0.001**
Diastolic blood pressure (mmHg)	66.88 ± 7.29	65.57 ± 7.32	69.16 ± 6.72	**0.001**
Mean arterial pressure (mmHg)	80.53 ± 8.24	78.11 ± 7.98	84.74 ± 6.94	** < 0.001**
Ocular perfusion pressure (mmHg)	43.52 ± 5.64	41.86 ± 5.50	46.37 ± 4.72	** < 0.001**
Pulse (beats per minute)	70.22 ± 10.45	70.43 ± 9.71	69.86 ± 11.75	0.778

Hemodynamic characteristics	Overall (N = 118)	Female (N = 75)	Male (N = 43)	P value
Peak velocity (cm/s)	10.49 ± 1.91	10.64 ± 1.84	10.23 ± 2.02	0.134
End diastolic velocity (cm/s)	3.49 ± 0.78	3.61 ± 0.81	3.27 ± 0.67	**0.025**
Time-averaged velocity (cm/s)	5.88 ± 1.18	6.07 ± 1.22	5.54 ± 1.04	**0.014**
Pulsatility index	1.21 ± 0.20	1.17 ± 0.20	1.26 ± 0.19	**0.014**
Resistive index	0.67 ± 0.05	0.66 ± 0.06	0.68 ± 0.05	0.060
Systolic/diastolic ratio	3.09 ± 0.54	3.03 ± 0.54	3.21 ± 0.54	0.065

Morphological parameters	Overall (N = 115)	Female (N = 72)	Male (N = 43)	P value
Total diameter (µm)	122.01 ± 11.78	121.90 ± 13.12	122.19 ± 9.26	0.889
Lumen diameter (µm)	98.51 ± 11.34	98.61 ± 12.68	98.33 ± 8.78	0.891
Average wall thickness (µm)	11.75 ± 1.44	11.65 ± 1.35	11.93 ± 1.58	0.321
Wall cross sectional area (µm^2^)	4069.14 ± 709.83	4028.60 ± 731.53	4137.02 ± 674.85	0.430
Wall to lumen ratio	0.24 ± 0.04	0.24 ± 0.04	0.24 ± 0.04	0.629

N, number of eyes. All parameters are summarized as mean ± standard deviation. P value, difference between genders.

Hemodynamic measures of CRA were obtained for all subjects with test–retest repeatability examined for 30 eyes. No significant differences between the two sessions were observed for any of the parameters. An intra-class correlation coefficient ranged from 0.815 to 0.981, and a coefficient of variation between 5 and 10% confirmed the good repeatability. As shown in Table 1, females had significantly higher end diastolic velocity (3.61 ± 0.81 vs. 3.27 ± 0.67 cm/s, p = 0.025), higher time-averaged mean velocity (6.07 ± 1.22 vs. 5.54 ± 1.04 cm/s, p = 0.014), and a lower pulsatility index (1.17 ± 0.20 vs. 1.26 ± 0.19, p = 0.014). Other hemodynamic parameters showed no significant gender difference.

Morphological data were available for 115 eyes. Three eyes were excluded due to unavailable measurement between 0.5 and 1 DD, from either the superior or inferior temporal branch. Most morphological assessment came from the superior temporal branch (N = 94) and the remainder from the inferior branch (N = 21). A subgroup analysis (35 eyes with both branches measured) was conducted to confirm the homogeneity of superior and inferior measurements. Paired t-tests revealed no significant inter-branch differences for all vessel parameters. Thus, measurements from the 21 inferior branches were combined with 94 superior branches to represent the morphological data of CRA branches. As suggested in Table 1, none of the AO parameters showed significant gender difference.

**Figure 3:**
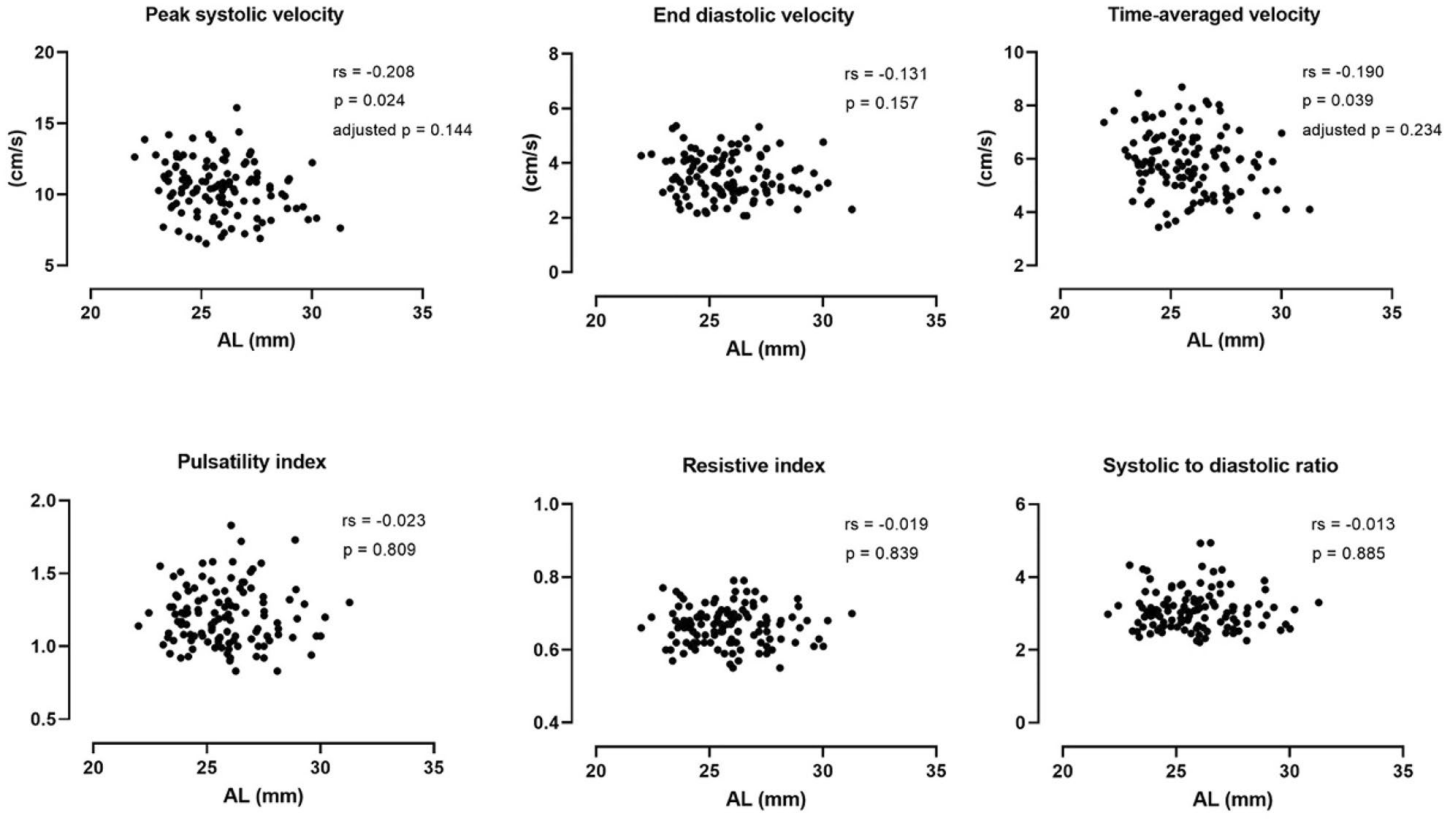
Bivariate Spearman correlations between axial length and hemodynamic parameters of the central retinal artery. Spearman correlation coefficients (rs), p-values and adjusted p-values after Bonferroni correction are presented.

Hemodynamic parameters of CRA were plotted against the AL and bivariate Spearman correlation analysis conducted (Fig. 3). The peak systolic velocity (rs = − 0.208, p = 0.024) and time-averaged velocity (rs = − 0.190, p = 0.039) were found to be mildly correlated with axial length. However, none of the correlations remained significant after adjusting the p-value for multiple tests. The end diastolic velocity and three resistance indices (PI, RI and S/D ratio) were all independent to axial elongation.

**Figure 4:**
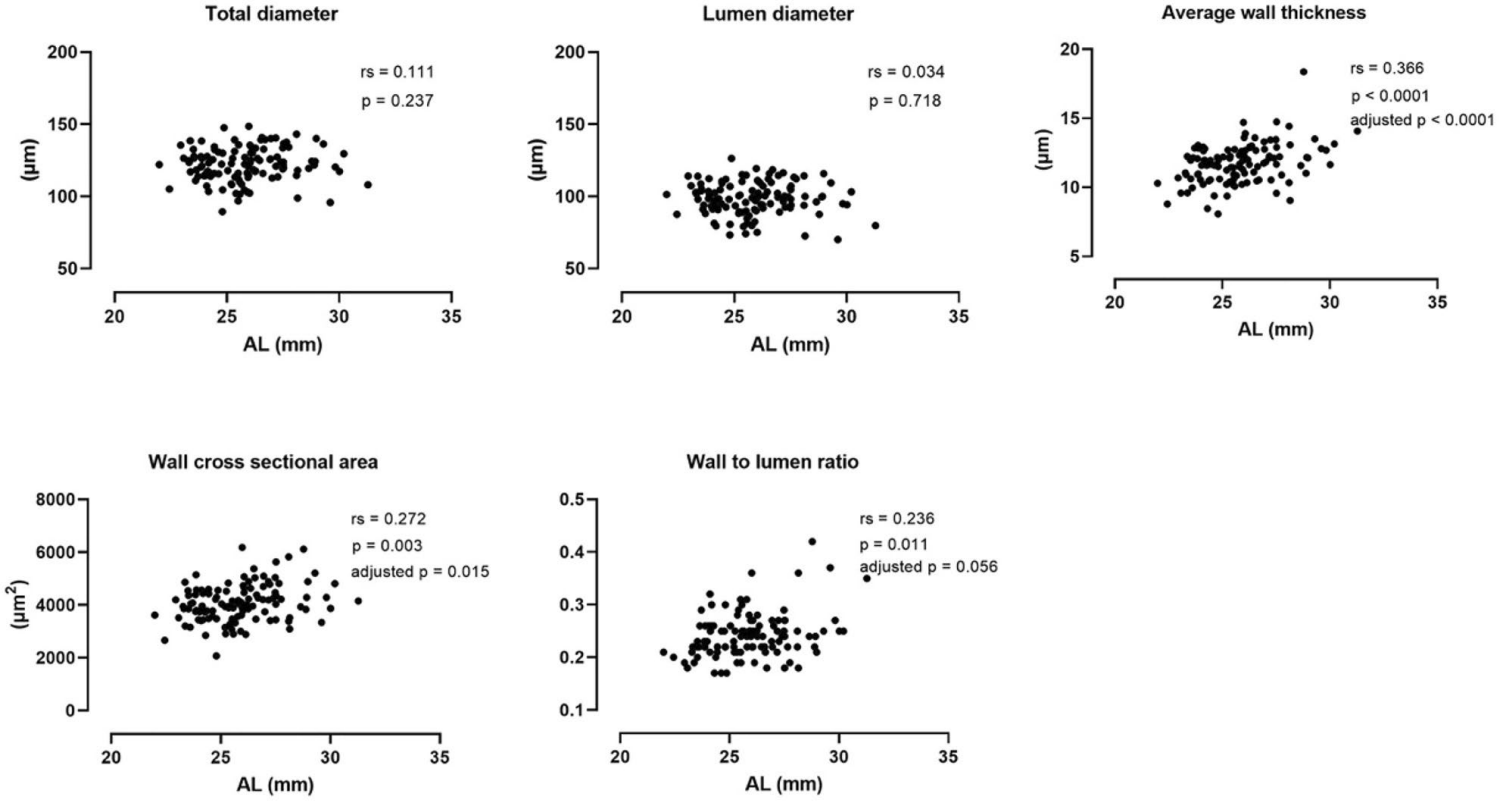
Bivariate Spearman correlations between axial length and morphological analysis of retinal artery. Spearman correlation coefficients (rs), p-values and adjusted p-values after Bonferroni correction were presented.

Figure 4 illustrates the correlation between morphological vessel parameters and axial length. Neither the total diameter nor the lumen diameter changed with increasing axial length, while the wall thickness (rs = 0.336, p < 0.001), wall cross sectional area (rs = 0.272, p = 0.003), wall to lumen ratio (rs = 0.236, p = 0.011) were significantly greater in longer eyes. Of these, the wall thickness and wall cross sectional area maintained positive associations with axial length after Bonferroni correction. Univariate linear regression further revealed that every 1 mm increase in axial length led to a 0.309 µm thickening of the wall thickness (F_(1, 113)_ = 20.55, R^2^ = 0.154, p < 0.001) and a 107.1 µm^2^ increase of wall cross sectional area (F_(1, 113)_ = 9.377, R^2^ = 0.077, p = 0.003).

## Discussion

This study analyzed the hemodynamic and morphological changes of the CRA in a large sample of myopic eyes with a wide range of refractive errors. In the retrobulbar segment, the CRA had a tendency of a slightly reduced flow velocity in eyeballs with longer axial length, but the correlations were not significant after adjusting for the multiple correlations. Vascular resistance was not affected by axial elongation, implying that the blood flow entering the inner retina may be similar for patients with low or high myopia. With respect to the intraretinal branches, the total diameter and lumen diameter of CRA branches did not have significant changes in longer eyes, although the wall thickness and the wall cross-sectional area both significantly increased, and the wall to lumen ratio marginally increased with increasing axial length. This implies some potential small artery remodeling in the intraretinal CRA branches of myopic subjects.

The CRA measurements in the current study fell into the range reported by previous findings^14–16,20^. Velocity measurements showed high inter-study variabilities, with a systolic velocity from 6.64 ± 1.98^14^ to 13.4 ± 3.3 cm/s^20^, a diastolic velocity from 2.01 ± 0.73^14^ to 4.4 ± 3 cm/s^15^, and a mean velocity from 3.34 ± 1.10^14^ to 16 ± 4 cm/s^16^. However, resistance indices were relatively comparable across studies. Resistive index was commonly reported with a mean value between 0.64 and 0.71^14,15,20^, which was similar to the 0.67 found in the current study. The pulsatility index and systolic to diastolic differences were rarely reported in previous studies.

Although the flow velocity and vascular resistance of the retrobulbar CRA remained unchanged in longer eyes, it remains unknown whether the arterial inflow of the inner retina was maintained since the vessel diameter of the retrobulbar CRA could not be determined by CDU. The diameter of the retrobulbar CRA is usually obtained from post-mortem analysis, with an average diameter of 0.36 mm^34^. Dorner, et al.^35^ proposed an alternative approach to estimate the retrobulbar CRA diameter based on the calculation of total retinal blood flow. The latter was obtained based on the velocity and diameter measurements of all veins entering the optic disc (i.e. branches of central retinal vein). However, the author specifically pointed out that this approach might not be applicable to longer eyes, as the value of venous diameter (i.e. in µm) was only available for in emmetropic eyes. Thus, estimating the ocular blood inflow through the CDU remains an unsolved difficulty.

Vessel diameters of the intraretinal CRA branches have been analyzed extensively by different imaging techniques, including fundus photo^23^, laser velocimeter^22^, and retinal oximeter^10^. Shimada et al.^10^ reported a vessel diameter of 104–128 µm at a distance of one to two DD from the disc, which was close to the total diameter obtained in current study (Table 1, 122.01 ± 11.78 µm). It is worthwhile mentioning that the vessel diameter in previous studies mostly referred to the total diameter, i.e. the sum of the lumen and wall thickness. However, the total diameter alone might not provide sufficient information about the changes in vessel structure. As suggested by Ueno et al.^36^, the total diameter could be maintained while artery remodeling occurred. Compared with healthy controls, eyes with proliferative diabetic retinopathy had a similar total diameter, but significantly reduced lumen diameter and increased wall thickness^36^, which indicated a decrease in the retinal blood flow. Therefore, extending the observation to vascular microstructures helps to capture the subtle changes in pathological conditions.

In myopic populations, artery remodeling may occur, as indicated by the increased wall thickness and wall cross-sectional area in the current study. However, the lumen diameter was not affected by axial elongation. Provided that the flow velocity of the CRA branches was comparable in emmetropes and high myopes as suggested by Shimada et al.^10^, the perfusion of the inner retina is likely to be preserved. Because the wall thickness only contributes to about 10% of the total diameter (Table 1), the increase in wall thickness may not result in a remarkable change in the total diameter. A similar explanation also applies to the wall to lumen ratio, which showed marginally significant elevation with increasing eyeball elongation.

It has been found that small artery remodeling occurred in hypertensive^37,38^ and diabetic patients^36,39^. An increased wall to lumen ratio might be the result of wall thickening, narrowing of the lumen, or the combination of both, which could be caused by vascular stenosis, vascular fibrosis, or growth of smooth muscle cells^36–39^. In general, there are two types of small arterial remodeling, eutrophic and hypertrophic^40^. The eutrophic remodeling is an inward procedure, which is characterized as reduced lumen and total diameter, and increased wall to lumen ratio. This involves the reorganization and migration of the vessel wall, without actual change in the wall tissue^40^. As the lumen diameter was independent to axial elongation in our young myopic population, so the arterial remodeling is unlikely to be this type. On the other hand, the hypertrophic remodeling, which is characterized as simultaneously increased wall to lumen ratio and wall cross sectional area, as a consequence of vascular smooth cells’ hypertrophy and hyperplasia^40^. This type of remodeling is triggered by a disordered retinal autoregulation which may happen without any underlying diseases, but as a response to difference stimuli, such as cold temperature, emotional or mechanical stress^41^. Therefore, we speculated that the excessive mechanical stress might be the trigger of arterial remodeling in myopic eyes. However, this hypothesis awaits to be confirmed by future study.

In the meantime, morphological parameters of vascular microstructure could be potential biomarkers to monitor myopia progression and improve our understanding of myopia-related vascular abnormalities in future studies. Given that recruited subjects in current study were all young and healthy myopes, the effect of ageing on these vascular characteristics was not examined. It is possible that more significant vascular abnormalities may be present in middle-age and older myopes. Because of the combined effects of age and high myopia, patients with vascular abnormalities may be more susceptible to a higher risk of developing myopic-related pathologies, such as myopic macular degeneration and choroidal neovascularization.

The current study does have some limitations. First, CRA measurements in this study mainly reflected the blood supply of the peripapillary region, but barely reached the macular area, where the key features of pathological myopic maculopathies occur. It is possible that the macular blood supply was affected as suggested by the impaired capillary perfusion^4,5^ and the enlarged foveal avascular zone^8,9^, even though the blood flow of the inner retina was maintained based on the current findings. Second, the morphological and hemodynamic measurements were not obtained on the same day. The mean day interval of the two sessions was 3.7 ± 3.7 days. Although the separated sessions were originally arranged to facilitate the data collection for test–retest repeatability of hemodynamic measures, unequal intervals among participants could be a possible confounder in data analysis. Third, the measurement time point of vascular morphologies during pulse cycles was not standardized. As pointed out by Knudtson, et al. ^42^, the caliber of retinal vessels varies with pulse cycle. Taking photography at a random point in the pulse cycle might introduce unrecognized source of variation and bias the direction of association. Especially for the small arteriole, there was a 20% difference between the minimum and maximum vessel diameters over the cycle. Future studies should consider standardizing the measurement time point, by synchronizing the image capturing with the pulse cycle, for more precise measurements. Lastly, blood pressure measurements were obtained from the left arms for all participants to calculate the mean arterial pressure and ocular perfusion pressure. This led to occasional minor mismatches between tested eyes (63 right and 55 left eyes) and ocular perfusion pressure. Ideally, the blood pressure should be measured on the same side with tested eye, although the arm-to-arm difference is very minimal in the normal population^43^.

## Conclusion

In normally-sighted young and healthy myopes, retrobulbar hemodynamics of the central retinal artery were unaffected by their eyeball elongation. The intraretinal branches of the central retinal artery showed subtle artery remodeling, as suggested by the thickening of vessel wall, however, the perfusion of the inner retina was likely to be maintained since the arterial lumen was unchanged.
