# Author Correction: Hemodynamic and morphological changes of the central retinal artery in myopic eyes

**DOI:** 10.1038/s41598-022-12902-1

**Published:** 2022-05-23

**Authors:** Mei Zhao, Andrew Kwok-Cheung Lam, Michael Tin-Cheung Ying, Allen Ming-Yan Cheong

**Affiliations:** 1 School of Optometry, Faculty of Health and Social Science, The Hong Kong Polytechnic University, Hung Hom, Kowloon, Hong Kong China; 2 Research Centre for SHARP Vision, The Hong Kong Polytechnic University, Hung Hom, Kowloon, Hong Kong China; 3 Centre for Eye and Vision Research, Kowloon, Hong Kong China; 4 Department of Health Technology and Informatics, The Hong Kong Polytechnic University, Hung Hom, Kowloon, Hong Kong China

Correction to: *Scientific Reports* 10.1038/s41598-022-11087-x, published online 02 May 2022

The original version of this Article contained an error in Affiliation 2, which was incorrectly given as ‘Centre for SHARP Vision, The Hong Kong Polytechnic University, Hung Hom, Kowloon, Hong Kong, China’. The correct affiliation is listed below:

Research Centre for SHARP Vision, The Hong Kong Polytechnic University, Hung Hom, Kowloon, Hong Kong, China

The original Article has been corrected.

